# Methyl group reorientation under ligand binding probed by pseudocontact shifts

**DOI:** 10.1007/s10858-018-0190-5

**Published:** 2018-06-02

**Authors:** Mathilde Lescanne, Puneet Ahuja, Anneloes Blok, Monika Timmer, Tomas Akerud, Marcellus Ubbink

**Affiliations:** 1 Leiden Institute of Chemistry, Leiden University, Einsteinweg 55, 2333 CC Leiden, The Netherlands; 2 Structure, Biophysics & Fragment-Based Lead Generation, Discovery Sciences, IMED Biotech Unit, AstraZeneca, Gothenburg, Sweden

**Keywords:** Isotope labeling, Pseudocontact shift, Methyl groups, NMR spectroscopy, Paramagnetic tag, Heat shock protein

## Abstract

Liquid-state NMR spectroscopy is a powerful technique to elucidate binding properties of ligands on proteins. Ligands binding in hydrophobic pockets are often in close proximity to methyl groups and binding can lead to subtle displacements of methyl containing side chains to accommodate the ligand. To establish whether pseudocontact shifts can be used to characterize ligand binding and the effects on methyl groups, the N-terminal domain of HSP90 was tagged with caged lanthanoid NMR probe 5 at three positions and titrated with a ligand. Binding was monitored using the resonances of leucine and valine methyl groups. The pseudocontact shifts (PCS) caused by ytterbium result in enhanced dispersion of the methyl spectrum, allowing more resonances to be observed. The effects of tag attachment on the spectrum and ligand binding are small. Significant changes in PCS were observed upon ligand binding, indicating displacements of several methyl groups. By determining the cross-section of PCS iso-surfaces generated by two or three paramagnetic centers, the new position of a methyl group can be estimated, showing displacements in the range of 1–3 Å for methyl groups in the binding site. The information about such subtle but significant changes may be used to improve docking studies and can find application in fragment-based drug discovery.

**Electronic supplementary material:**

The online version of this article (10.1007/s10858-018-0190-5) contains supplementary material, which is available to authorized users.

## Introduction

Fragment-based drug discovery (FBDD) has proven to be an effective method to develop medicinal drugs (Erlanson 2012). FBDD is based on finding very small molecules that bind to the target with a large average binding energy per heavy atom (~ 0.3 kcal/mol/heavy atom). Such fragments still have a low affinity and then need to be elaborated to molecules with more negative binding free energies, either by linking fragment hits or by growing them (Bohacek et al. 1996; Hajduk and Greer 2007). For the elaboration of hits into lead compounds FBDD depends heavily on structural analysis of fragment-target complexes by X-ray diffraction of crystals or NMR spectroscopy. The former technique is most commonly used but NMR is an alternative for structure determination and offers complementary information. Structure determination of the complex by NMR requires a complete NOE analysis of protein and ligand, which is tedious but can be used in cases where X-ray crystallography fails (Pellecchia et al. 2008). Other, less demanding methods are based on transferred NOEs, paramagnetic relaxation enhancements (PRE) or pseudocontact shifts (PCS) to obtain information about the ligand bound state while benefitting from the narrow linewidths of the ligand in the free state (Viegas et al. 2011; Guan et al. 2013; Jahnke et al. 2000, 2001).

We aim to investigate the possibilities of PCS to study ligand–protein interactions. PCS have been used before to obtain model of ligands bound to proteins (Guan et al. 2013; Tu and Gochin 1999; Saio et al. 2011; John et al. 2006). Before, we demonstrated that ligands that are in fast exchange between bound and free state can exhibit transferred PCS caused by lanthanoid tags on the protein, which can be used to determine a low-resolution model of the ligand in the binding site, provided that a structure of the protein is available and under the assumption that ligand binding does not result in backbone conformational changes. Fragments, as well as larger compounds often bind in hydrophobic pockets on the protein where they do not alter the positions of backbone atoms significantly. Methyl groups are often found in such pockets and, thus, are in direct contact with the ligand. They are prone to experience chemical shift changes due to changes in the chemical environment upon ligand binding, and may also more readily than backbone atoms show conformational changes due to the rotational freedom of side-chains (Wiesnerl and Sprangers 2015). In this way, they can help to accommodate ligand binding, enabling it to form optimal interactions. Therefore, we wondered whether such changes could be detected by using PCS. These shifts are caused by the interaction of the nuclear spin and the spin of unpaired electron(s) in a paramagnetic center. They depend on the distance between the spin and the center to the third power as well as on the orientation of the spin in the frame of the anisotropic component of the magnetic susceptibility of the unpaired electron(s) (Otting et al. 2010; Liu et al. 2014). With a probe that is rigid relative to the protein and a proper diamagnetic control, the PCS can be predicted and measured with very high accuracy and small changes in the location of the spin relative to the paramagnetic center can result in measurable PCS changes. Methyl PCS can be observed in sensitive 2D NMR spectra, potentially also on large systems by applying selective labelling in a deuterated background and by using TROSY-based experiments (Tugarinov et al. 2003; Tugarinov and Kay 2005; Sprangers et al. 2008).

Here, we describe the use of PCS as structural restraints to probe at the same time binding kinetics and structural changes of the protein ntd-HSP90 upon fragment binding. HSP90 is a target protein against cancer (Nagaraju et al. 2017) and its ATP binding site located in the N-terminal domain (ntd) is targeted for inhibition (Li et al. 2012). HSP90 is a molecular chaperone essential to prevent client proteins from ubiquitin–proteasome system degradation. More than 200 client proteins of HSP90 have been identified, including oncoproteins (Murray et al. 2010). Therefore, HSP90 is a cancer-target protein and inhibitors have been found to bind the N-terminal domain and/or the C-terminal domain (Den and Lu 2012). Several potent molecules are clinical candidates for cancer treatment through inhibition of the ATPase activity and FBDD has been successfully applied to HSP90, which led to a clinical trial (Murray et al. 2010). We find that ligand binding is only marginally affected by attaching the two-armed lanthanoid tag CLaNP-5 (Keizers et al. 2007, 2008) to ntd-HSP90 at three locations. Methyl group resonances show extensive chemical shift perturbations in the binding site, as well as further in the hydrophobic core of the protein. Several significant PCS changes are observed upon ligand binding, which can be interpreted as movements of the methyl groups of a few Ångström. These changes can be translated into structural restraints that may be used in ligand docking studies.

## Materials and methods

### Sample preparation

Three double cysteine mutants of the ntd-HSP90 were designed on the surface of the protein, S50C/D54C, A101C/N105C and T149C/I187C (Lescanne et al. 2017). Ntd-HSP90 does not have any native cysteines. The protein was produced labelled with Leu-δ1-δ2/Val-γ1-γ2-[^13^CH_3_] and purified and tagged with CLaNP5 according to a published protocol (Lescanne et al. 2017). CLaNP-5 was synthesized as described before (Keizers et al. 2007, 2008).

### NMR titration

**Fig. 1:**
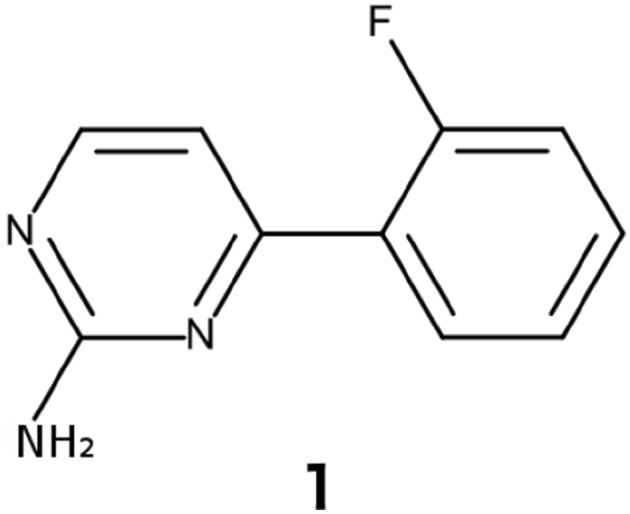
Structure of **1** used to titrate ntd-HSP90

Ntd-HSP90 in 50 mM Tris-HCl and 50 mM NaCl buffer, pH 7.7, was titrated with 4-(2-Fluorophenyl)-2-pyrimidinamine, **1**, (Fig. 1), a 189 Da known ligand of ntd-HSP90 that was kindly provided by AstraZeneca (Göteborg, Sweden). Titrations were performed with three ntd-HSP90 mutants, S50C/D54C, A101C/N105C and T149C/I187C tagged with Lu^3+^-CLaNP-5 or Yb^3+^-CLaNP-5. The concentrations of S50C/D54C, A101C/N105C T149C/I187C were 20, 103 and 65 µM, respectively, for both diamagnetic and paramagnetic forms of the protein. Concentrations (µM) of **1** for titrations with S50C/D54C, A101C/N105C and T149C/I187C were [0, 39, 59, 88, 132, 198, 296, 444, 667, 1000], [0, 20, 40, 121, 364, 1111] and [0, 40, 89, 200, 442, 665, 1008, 1897], respectively. The NMR sample volume was 595 µL for all samples, and dilution was neglected, because the biggest volume of ligand solution added was < 5 µL. ^13^C-^1^H HSQC (Palmer et al. 1991; Kay et al. 1992; Schleucher et al. 1994) spectra were acquired at each titration point, on a Bruker Avance III 800 MHz spectrometer, equipped with a cryogenically cooled TXI-probe head, operating at 298 K. Spectra were processed with nmrpipe (Delaglio et al. 1995) using the exponential EM apodization function for analysis with TITAN (Waudby et al. 2016). A similar titration was performed with WT ntd-HSP90 observing the amide groups. Average CSP were calculated as (ΔH^2^ + (ΔC/10)^2^)^0.5^ for methyl groups and (ΔH^2^ + (ΔN/6)^2^)^0.5^ for amides. PCS were measured using ^1^H and for calculations the geometrical average of the proton coordinates in methyl groups were used.

### Assignments

Methyl groups assignments have been performed before with traditional through-backbone NMR techniques and confirmed by PARAssign (Skinner et al. 2013; Lescanne et al. 2017). PARAssign provided the stereo-specific assignment with high reliability for 14 Leu/Val methyl groups (Lescanne et al. 2017), based on PCS generated by CLaNP-5 attached at two distinct positions, S50C/D54C and A101C/N105C.

### Cross-section refinement

A home-written python script was used to define possible locations of the bound methyl groups locations. PCS iso-surfaces were calculated for a grid of 15 × 15 × 15 Å, with 100 points per dimension and centered on the methyl group position in the crystal structure of free ntd-HSP90 [PDB entry 3t0h (Li et al. 2012)]. Cross-sections of iso-surfaces from different tags were defined by finding the positions within the grid that matched the required PCS of all tags within an error of 0.02 ppm (0.03 ppm in one case, see below).

### Q and Qa factors

Q and Qa factors were used to quantify deviation between experimental and predicted data. Q and Qa were calculated according to Eqs. 1 and 2, respectively. Q is the usual measure for goodness of fit, Qa is, however, less sensitive to bias toward cases in which the predicted value is much larger than the observed one, as compared to the opposite case, in which the predicted value is much smaller than the observed one (Bashir et al. 2010). In cases of a good fit, Qa ≈ 0.5Q.

1 $$Q=\sqrt {\frac{{\mathop \sum \nolimits^{} {{\left( {\delta _{{PCS,i}}^{{ped}} - \delta _{{PCS,i}}^{{exp}}} \right)}^2}}}{{\mathop \sum \nolimits^{} {{\left( {\delta _{{PCS,i}}^{{exp}}} \right)}^2}}}}$$

2 $$Qa=\sqrt {\frac{{\mathop \sum \nolimits^{} {{\left( {\delta _{{PCS,i}}^{{pred}} - \delta _{{PCS,i}}^{{exp}}} \right)}^2}}}{{\mathop \sum \nolimits^{} {{\left( {\left| {\delta _{{PCS,i}}^{{exp}}} \right|+\left| {\delta _{{PCS,i}}^{{pred}}} \right|} \right)}^2}}}}$$

## Results

### Tagging effects

**Fig. 2:**
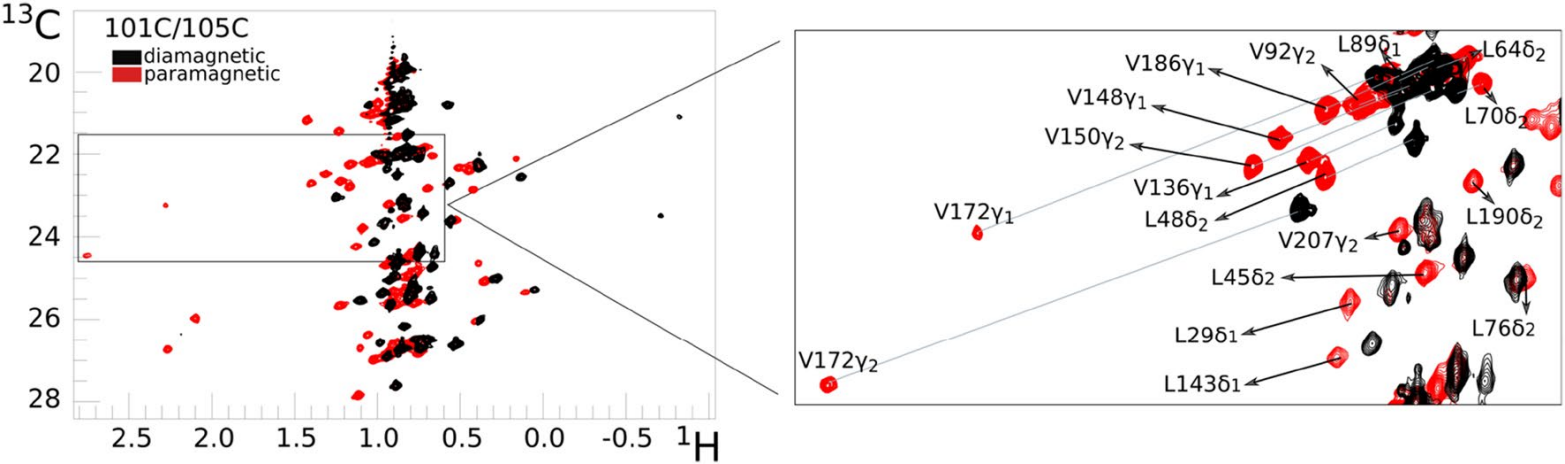
Enhancement of spectral dispersion by PCS. An overlay is shown of Leu/Val methyl HSQC spectra of ntd-HSP90101C/105C tagged with CLaNP-5 loaded with Lu^3+^ (black contours) or Yb^3+^ (red contours). The inset shows a detail and the lines connect equivalent resonances

Ntd-HSP90 was tagged at three sites using the Caged Lanthanoid NMR probe #5 (CLaNP-5), containing either Lu^3+^ as a diamagnetic control or Yb^3+^ as a paramagnetic center. The tagging sites, double mutants 50C/54C, 101C/105C and 149C/187C, have been described before (Lescanne et al. 2017). Assignments are shown in Fig. S1. Methyl ^13^C-^1^H HSQC spectra of WT and CLaNP-5(Lu^3+^) tagged mutants are very similar except for the resonance of a few methyl groups very close to the tags (Lescanne et al. 2017), indicating that the tags do not have large effects on the structure of the protein. A first comparison of the methyl group spectra of the paramagnetic and diamagnetic samples illustrates the increased dispersion of the resonances for the paramagnetic samples (Fig. 2), which has been noted before (Sattler and Fesik 1997). For example, a crowded spectral region with a width of 0.5 ppm in the ^1^H dimension in the spectrum of the diamagnetic sample, disperses over 1.0 ppm for mutant 50C/54C and up to 3.0 ppm for mutant 101C/105C in the spectrum of the paramagnetic sample. Increased dispersion is of interest for methyl group HSQC spectra, because the resonances are often more crowded than in amide HSQC spectra, in particular for Leu and Val methyl hydrogens and to a lesser extent methyl carbons. In principle, lanthanoids with larger paramagnetic effects (Tm^3+^, Dy^3+^, Tb^3+^) provide even more dispersion but also cause considerable paramagnetic relaxation farther from the metal than in the case of Yb^3+^. Thus, such lanthanoids are more appropriate to generate dispersion in spectra of bigger proteins.

**Fig. 3:**
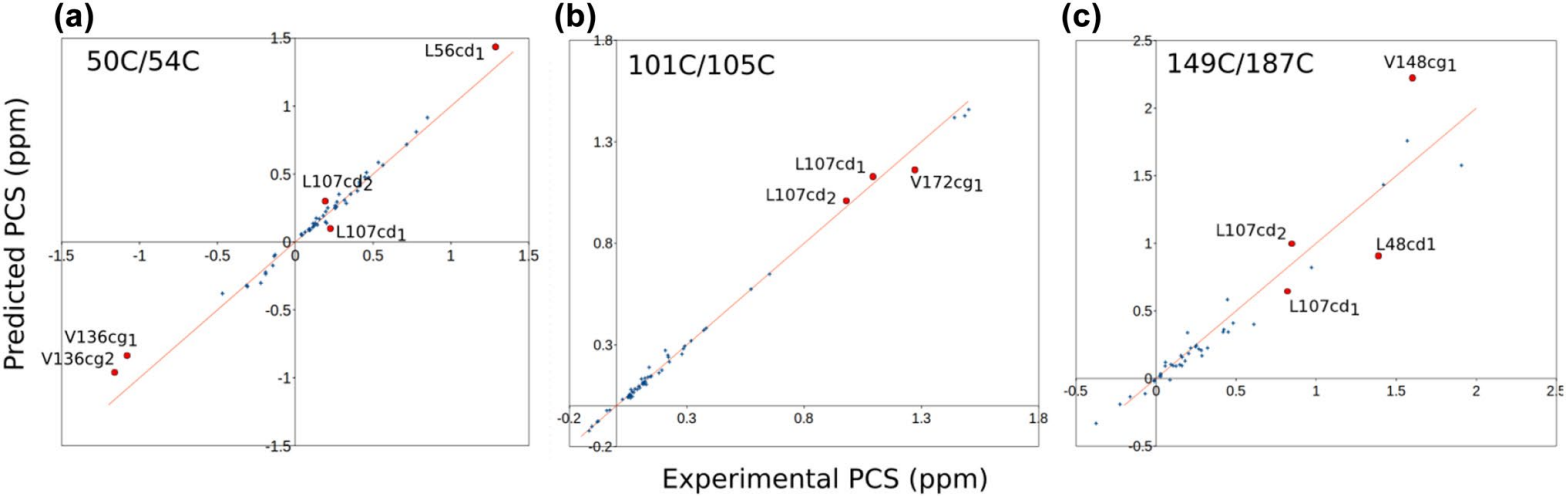
Prediction of methyl group PCS. The PCS for the Leu/Val methyl groups of ntd-HSP90 were predicted using the published amide based Δχ tensor parameters (Lescanne et al. 2017) and the structure with PDB entry 3t0h (Li et al. 2012) and plotted against the experimental PCS. No fitting was performed. **a** For mutant 50C/54C the Q factor (Eq. 1) is 0.14 (Q_a_ = 0.07, Eq. 2) **b** For mutant 101C/105C Q = 0.05 (Q_a_=0.025). **c** For mutant 149C/187C Q = 0.24 (Q_a_=0.12). The red line represents a perfect correlation

**Fig. 4:**
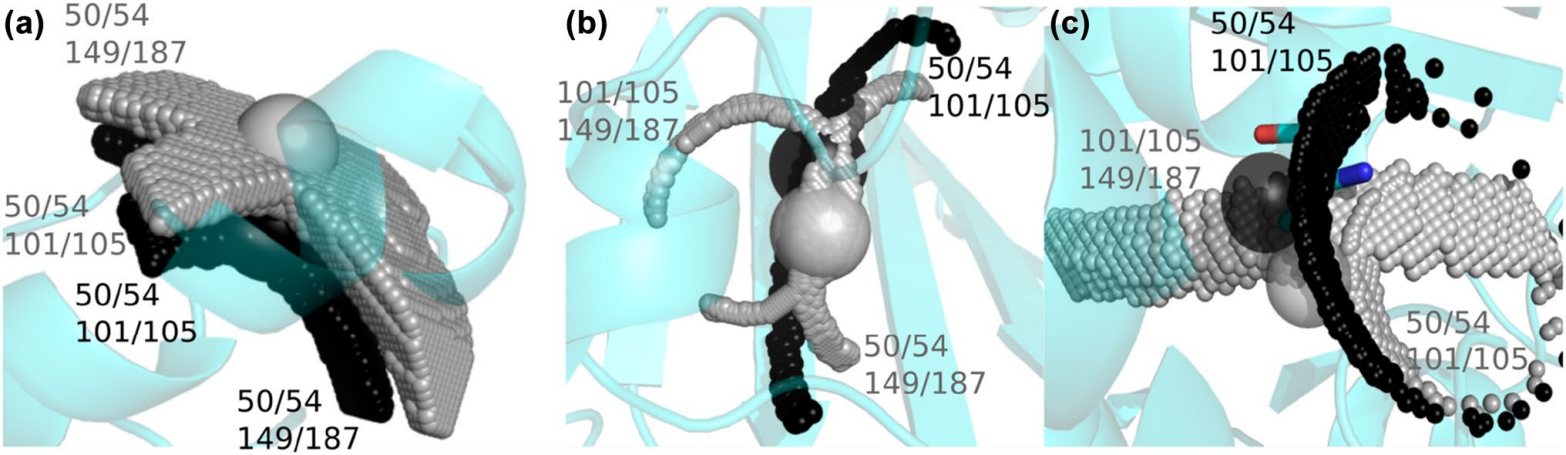
Cross-sections of iso-surfaces of experimental free PCS, with the free structure 3t0h. Large grey and black spheres represent the crystal structure locations of methyl groups, centred on the carbon methyl group with radius of 1 Å, for Valγ1/Leuδ1 and Valγ2/Leuδ2, respectively. Grey and black spheres represent the experimental PCS cross-sections for Valγ1/Leuδ1 and Valγ2/Leuδ2, respectively for the free ntd-HSP90. Each cross-section was calculated using 0.02 ppm error on the PCS, for a cubic grid with sides of 50 points over 5 Å, centred on the methyl group of interest. **a** Leu70 methyl groups. The two grey and black areas are cross-sections from mutant 50C/54C with mutant 101C/105C and mutant 50C/54C with 149C/187C for Leu70 δ1 and δ2, respectively. **b** Val150 methyl groups. The grey areas are the γ1 cross-sections from mutant 101C/105C with mutant 149C/187C and from mutant 50C/54C with 149C/187C, the black area is the V150 γ2 cross-section for mutant 50C/54C with mutant 101C/105C. **c** Val136 methyl groups. In black spheres the PCS iso-surfaces cross-section of mutant 50C/54C with mutant 101C/105C is shown for methyl γ2. In grey spheres are the two PCS iso-surface cross-sections of mutant 50C/54C with 101C/105C and mutant 101C/105C with mutant 149C/187C. Both crystal structure methyl groups are on the edge of the cross-section

Magnetic susceptibility (Δχ) tensors were refined previously using amide proton PCS (Lescanne et al. 2017). Methyl group PCS were predicted based on these tensors and the ligand-free structure [PDB entry 3t0h (Li et al. 2012)] and compared to the experimental ones. For mutants 50C/54C and 101C/105C most predicted PCS fit the experimental values well (Fig. 3). Mutant 149C/187C shows a poorer fit but that is in line with the results for the amides (Fig. S2), which was attributed to the fact that the tag crosslinks two β-strands and appears to assume two conformations (Lescanne et al. 2017). To illustrate how differences between experimental and predicted PCS translate into differences in the expected locations of the nuclei in the protein, cross-sections of experimental PCS iso-surfaces for the different mutants were calculated (Fig. 4). The iso-surface identifies all locations around a paramagnetic center with a given PCS. The cross-sections are the overlap areas of two or three iso-surfaces from different tag locations. A cross-section was calculated such that its thickness reflects 0.02 ppm uncertainty. Thus, large cross-sections (Fig. 4a) indicate a weak PCS gradient, as is observed far from the paramagnetic centers. Thin cross-sections (Fig. 4b) report on a steep PCS gradient, closer to the paramagnetic center or close to where the PCS changes sign. Cross-sections for the iso-surfaces of pairs of mutants are shown in Fig. 4, panels A and B, for the methyl groups from residues Leu70 and Val150. The predicted PCS of these methyl groups match the experimental PCS within 0.02 ppm. The methyl groups observed in the crystal structure are located at the intersection of the two cross-sections, i.e., at the position that matches the experimental PCS for all three mutants within 0.02 ppm. For some methyl groups the discrepancy between experimental PCS and PCS predicted on the basis of the crystal structure is larger than 0.02 ppm. For instance, the experimental PCS of Val136 γ1 and γ2 are − 1.03 and − 1.12 ppm, respectively, and the predicted values show deviations of 0.19 (− 0.84 ppm) and 0.16 ppm (− 0.96 ppm) for mutant 50C/54C for γ1 and γ2, respectively. Because of the strong PCS gradient, these large PCS deviations translate in only a small displacement of about 0.6 Å as compared to the crystal structure (Fig. 4c). In the case of the mutant 149C/187C, the experimental PCS of Val136 γ1 and γ2 equal 0.27 and 0.27 and the deviations from the predicted values are only 0.03 (0.23 ppm) and 0.05 ppm (0.22 ppm), respectively, yet these translate into sizeable displacements of 1.5 Å, due to the weak PCS gradient at this position. This remarkable difference is visualized in Fig. S3. Thus, these findings demonstrate that deviations larger than the measurement error of PCS (usually 0.02 ppm or less) can be caused by very subtle differences in structure, whereas small deviations may still reflect a more sizeable mismatch between observed PCS and the structure used for PCS prediction. The PCS gradient at the methyl group position can be used to evaluate the structural relevance for observed differences between experimental and predicted PCS. To establish to what degree the uncertainty in the Δχ tensor parameters affects the iso-surfaces, the Δχ tensors were also calculated using the methyl PCS as input, rather than the amide PCS. These independent data report on the same tensor, so differences are a measure for the uncertainty. The fitting involves eight parameters that are not completely uncorrelated, so slightly different solutions can be found. It was found that the difference are indeed small (Fig. S4). The effect on the calculated iso-surfaces only is significant for residues with a steep PCS gradient, Fig. S5.

**Fig. 5:**
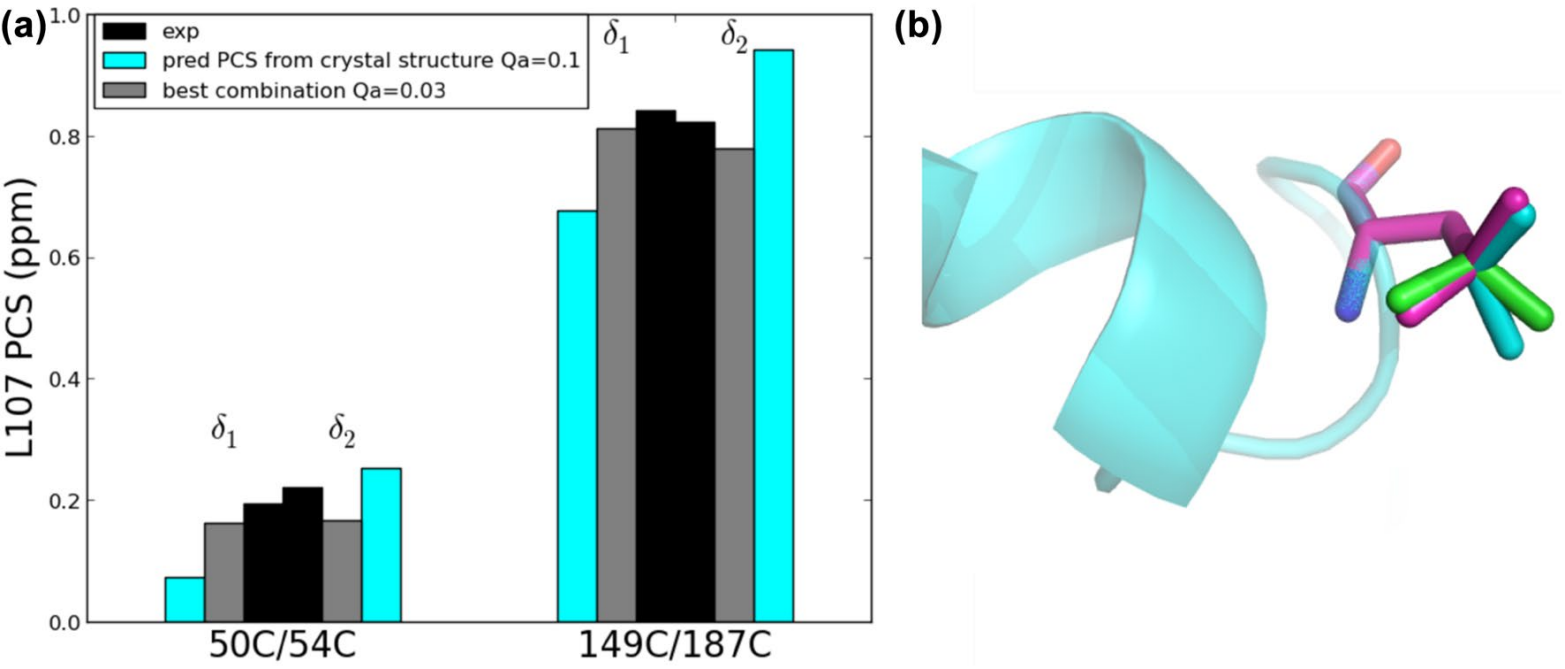
**a** Experimental (black) and predicted PCS for the two methyl groups of L107 for rotamer 2, observed in the crystal structure [PDB entry 3t0h (Li et al. 2012)] in cyan and for the combination of rotamer 2 and 3 in grey, for the two mutants 50C/54C and 149C/187C. **b** Residue L107. The rotamer found in the crystal structure is shown in cyan sticks. Rotamers 1 and 3 are in green and magenta, respectively. The rotamers were generated with Pymol (DeLano 2009). Note that the idealized, generated rotamers shows slightly different angles compared to the crystal structure rotamer

For residue Leu107, the predicted PCS correlated poorly with the experimental ones for mutants 50C/54C and 149C/187C. The predicted PCS are clearly distinct for the δ1 and δ2 methyl groups (0.072 and 0.252 ppm for mutant 50C/54C and 0.68 and 0.94 ppm for mutant 149C/187C), whereas the experimental values are very similar (0.221 and 0.194 ppm for mutant 50C/54C and 0.822 and 0.842 ppm for mutant 149C/187C). The similarity of the two values suggests a kind of averaging. The linewidth of the resonances in the diamagnetic sample also suggests a form of exchange. To establish whether population of more than one rotamer explains the deviating PCS, the populations of each of the three rotamers were determined. Each rotamer was modelled in the structure with Pymol (DeLano 2009) (ignoring steric clashes) and the PCS were predicted. The best fit was found for an exchange between two rotamers populated at 53 and 47%, with the third rotamer not being populated, Fig. 5. The Qa fit quality factor (see Eq. 2) for Leu107 drops from 0.14 to 0.03 when predicted PCS are calculated as a combination of rotamer 2 (+ 0°) and rotamer 3 (+ 120°), for mutants 50C/54C and 149C/187C. For mutant 101C/105C, rotamer 2 fits the data well and admixture of rotamer 3 reduces the fit quality. However, Leu107 is located very close to the tag in this mutant, and near a reported flexible region (Didenko et al. 2012), so either the methyl group location or the exchange populations of rotamers could be influenced by the CLaNP tag. It is interesting that the PCS seem to provide evidence for rotamer exchange and can be used to estimate populations. Such dynamics is not obvious from the chemical shift. Definitive evidence, however, would require further experiments, which were beyond the scope of this work. No other methyl groups were found to be experiencing the same phenomenon.

### Ligand titration

**Fig. 6:**
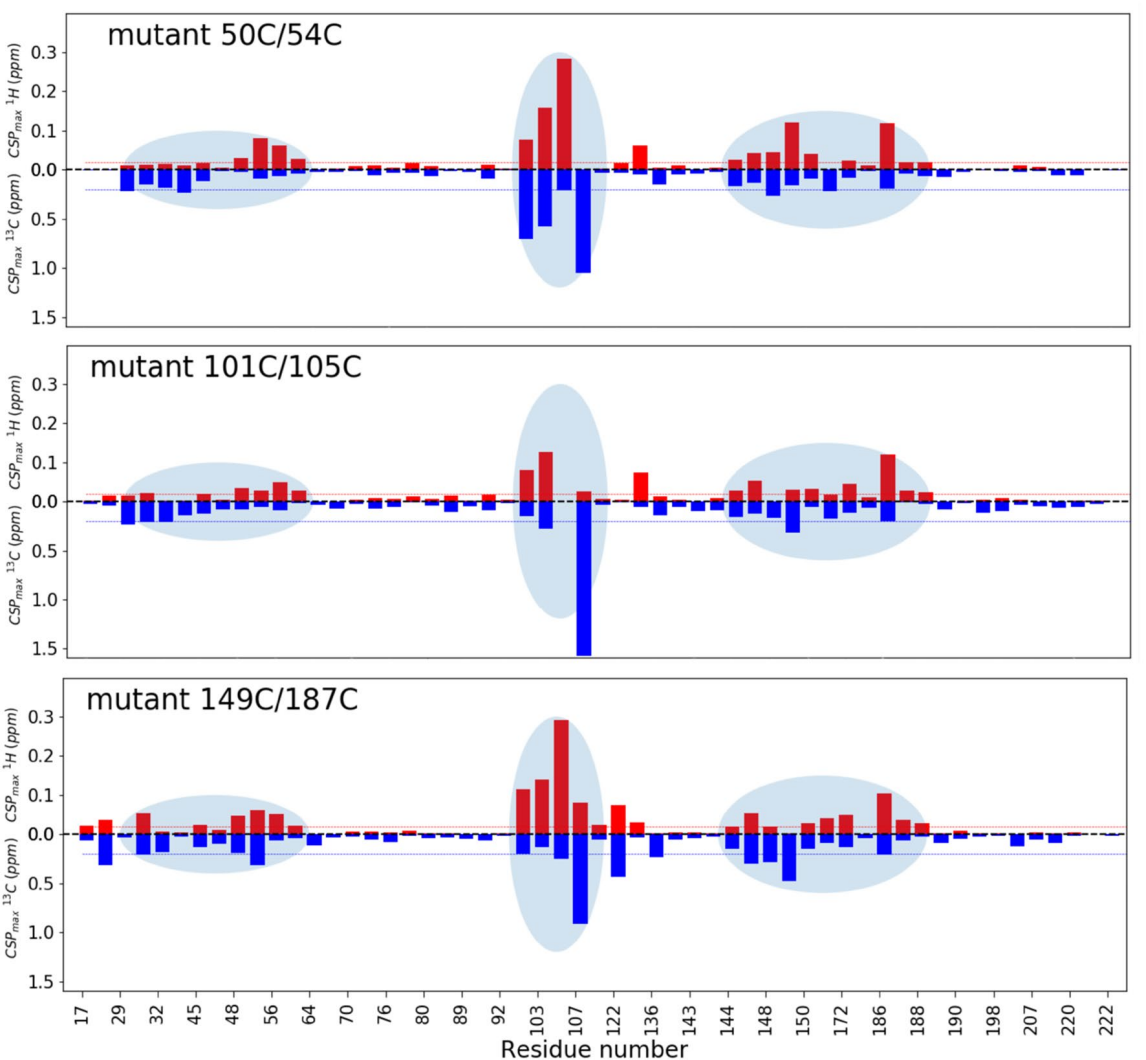
Ligand binding. |CSP| are plotted for methyl group resonances upon binding of **1**. The ^1^H and ^13^C CSP are shown in red and blue bars, respectively. The CSP have been extrapolated to the 100% bound state. The red and blue lines mark 0.02 and 0.2 ppm, respectively. The light blue ovals highlight the most perturbed methyl groups

**Fig. 7:**
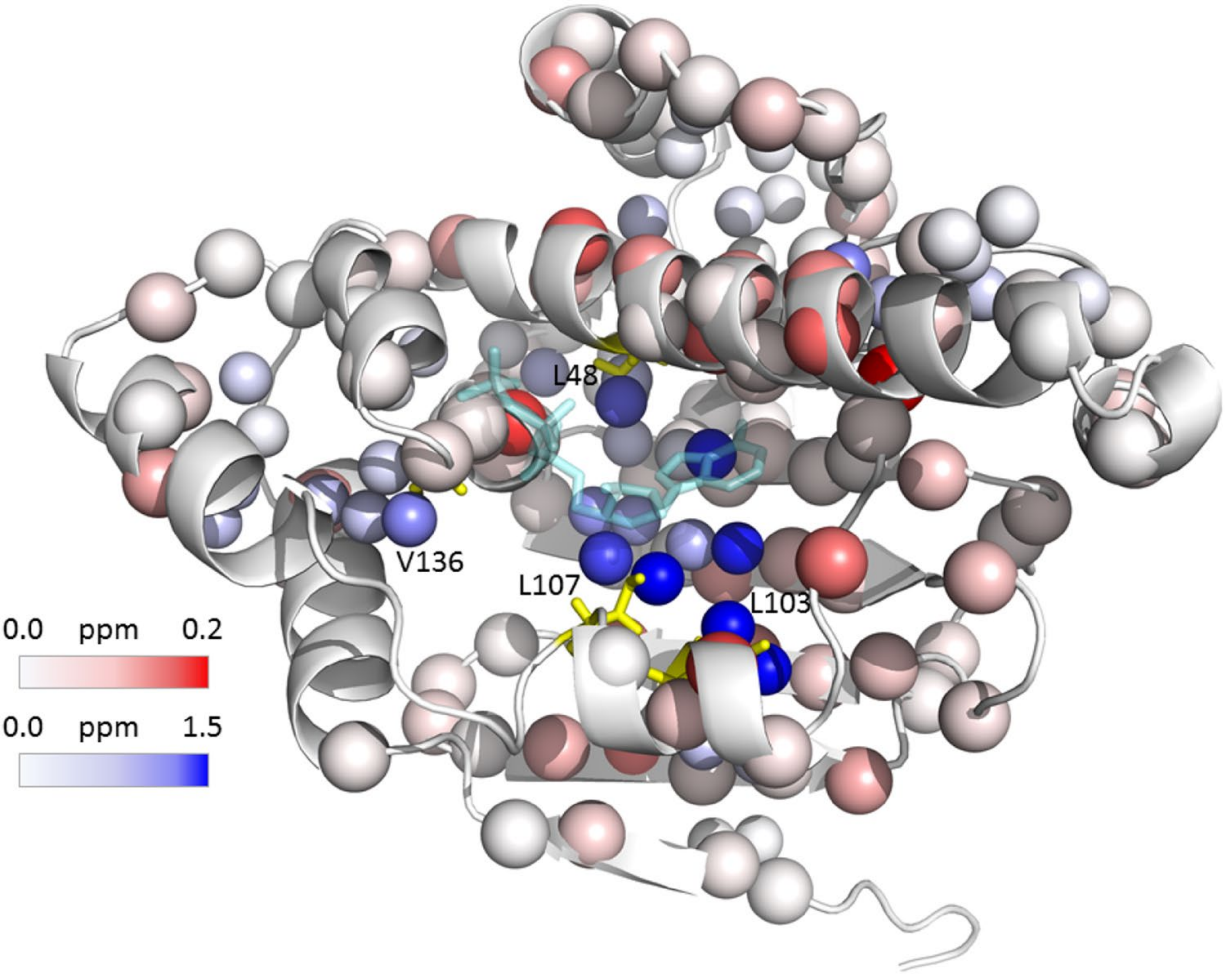
CSP map for binding of **1** to ntd-HSP90. The smaller spheres represent the Leu and Val methyl groups, coloured in a gradient of white to blue for increasing CSP as observed in the titration of mutant 50C/54C tagged with CLaNP-5 (Lu^3+^). The larger spheres represent the amide nitrogens that were observed in a titration of WT ntd-HSP90, coloured according the average amide CSP (from white to red). The residues L48, L103, L107 and V136, harboring a methyl group with |ΔPCS| > 0.04 ppm for at least two mutants, are shown in yellow sticks. The structure is taken from PDB entry 3t0z. The ATP ligand observed in that structure is indicated by semi-tranparent, cyan sticks to indicate the binding site

First, ntd-HSP90 mutants tagged with diamagnetic CLaNP-5 were titrated with the weakly binding ligand **1**. Three regions of the protein exhibit chemical shift perturbations (CSP) upon titration **(**Fig. 6), revealing a localized binding site, in a cleft of the protein, but with small CSP being observed relatively far away in the core of the protein, Fig. 7. The affected regions are similar for the three mutants (Fig. 6), suggesting that the tag at different locations does not alter the binding location. Moreover, most peaks show similar CSP directions in the two-dimensional HSQC spectra (Fig. S6), indicating similar changes in the chemical environment of the methyl groups upon ligand binding. An exception is the resonance for Leu103 δ1, for which the CSP is not the same for all mutants. The discrepancy is largest for mutant 101C/105C, in which the Leu is very close to the tag, being located in between the two engineered Cys residues. Thus, the tag at this position appears to have some effect on ligand binding. A titration of the WT ntd-HSP90 in which the amide groups were observed was also performed. The results are in line with the methyl titrations, with the largest effects near the binding cleft, in particular in the long α-helix that lines the binding site. Also for the amides, smaller effects are seen far from the binding site, suggesting that binding effects are transmitted into the rest of the protein (Fig. 7).

### Binding parameters

The dissociation constant K_D,_ as well as the dissociation rate constant (k_OFF_) were calculated with TITAN software (Waudby et al. 2016) using a 1:1 binding model (A + B ⇆ A − B). TITAN fits both the equilibrium constant (K_D_) and the dissociation rate constant (k_OFF_) on the basis of the CSP and line broadening. A global fit of the resonances of five methyl groups (Table S1) was performed and then one at the time was taken out and the fit repeated. The WT K_D_ was calculated using a titration performed on the ^15^N labelled protein, on the basis of four amide protons (Table S1). The ranges of values obtained in this way are reported in Table 1 and provide a more realistic error range than the fit error. Differences are observed between the different mutants, suggesting that the affinity is influenced by the tags to some degree, in particular for mutant 149C/187C. K_D_ values of 200 and 150 µM were used for the calculation of the 100% bound state CSP for mutant 50C/54C and 101C/105C respectively. For these two mutants, a 50 µM variation of the K_D_ results in a change of the extrapolated CSP of 0.01 ppm at most, half the error used for the further calculations. For mutant 149C/187C a K_D_ of 50 µM was used to extrapolate the CSP.

**Table 1:** Parameters for binding of **1** to ntd-HSP90 derived from the titrations with TITAN software, for the WT and three mutants

	K_D_ (µM)	k_OFF_ (s^−1^)
WT	160–220	1400–1800
50C/54C	195–238	1500–2300
101C/105C	140–171	1075–1300
149C/187C	41–45	1300–1900

### Methyl group re-orientation

**Fig. 8:**
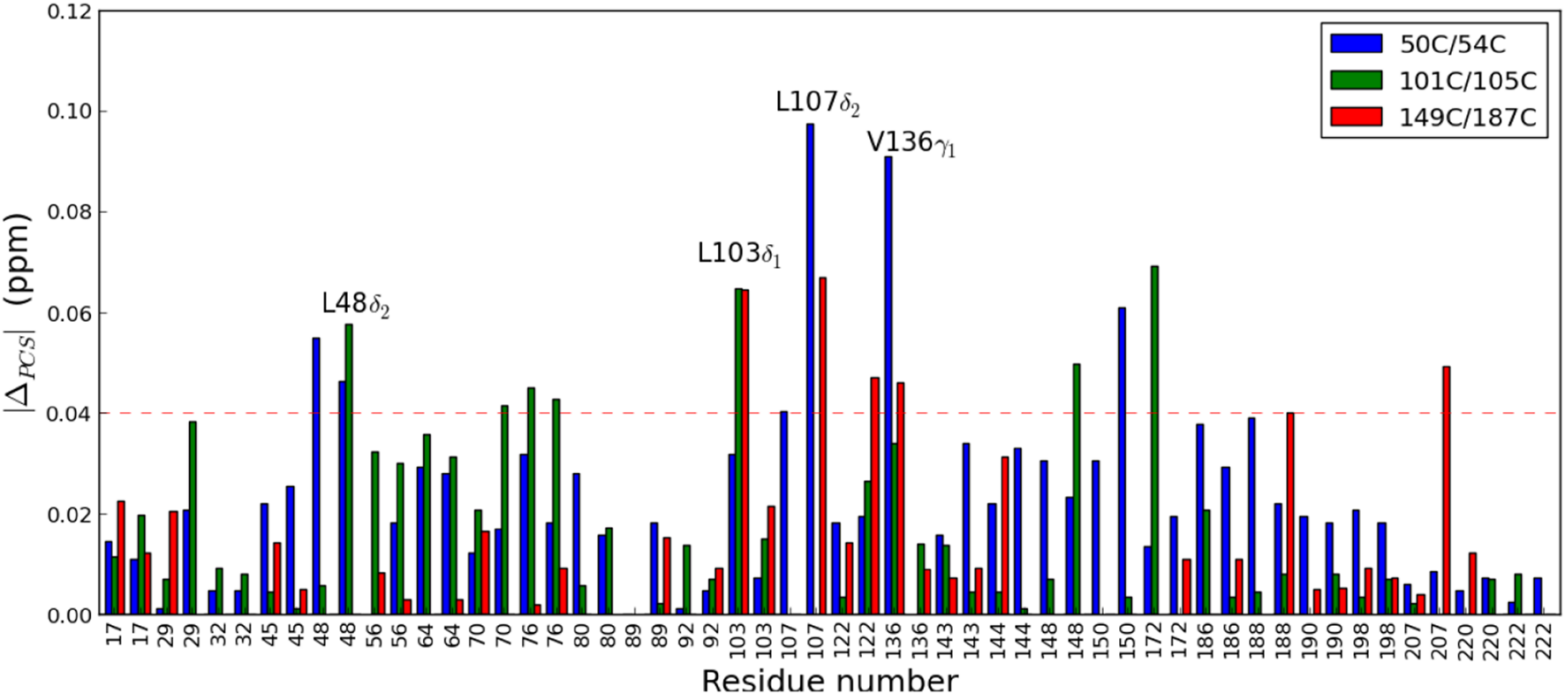
PCS changes upon ligand binding. |ΔPCS| for Leu/Val methyl groups for the bound state are shown as blue, green and red bars for the mutants 50C/54C, 101C/105C and 149C/187C, respectively. The red dashed line represents the threshold of 0.04 ppm, based on the estimated error. The conservative maximum experimental error in the chemical shifts is estimated to be 0.02 ppm. The propagated error in ΔPCS is thus 0.04 ppm

The titrations were repeated with ntd-HSP90 tagged with CLaNP-5 (Yb^3+^). The binding characteristics were the same as for the diamagnetic sample. To obtain the PCS for the ligand bound state, the maximal CSP, representing ntd-HSP90 fully saturated with **1**, were calculated for both diamagnetic and paramagnetic proteins by extrapolation from the CSP data in the last point of the titration, by using the K_D_ values. The extrapolated CSP were subtracted to obtain the PCS of the bound state. Ligand binding causes changes in some PCS up to 0.1 ppm, Fig. 8. All PCS values are provided in Table S2. Methyl groups exhibiting significant ΔPCS, larger than 0.04 ppm for at least two of the paramagnetic centers, were selected for further analysis, comprising L48_δ2_, L103_δ1_, L107_δ2_ and V136_γ1_ (Table 2). These residues surround the binding site of ntd-HSP90, Fig. 7. Changes in PCS could be caused by changes in the position of the methyl groups due to interactions with the ligand, by changes in the tag position or orientation or by a combination of both effects. The Δχ tensors were calculated from the PCS of the complex of ntd-HSP90 and **1** (Table S3) and back-predicted PCS were compared with those of the free protein. The correlation is very good, with Qa values of 0.03, 0.04 and 0.16 for mutants 50C/54C, 101C/105C and 149C/187C, respectively (Fig. S7). The Qa values are very similar to those found between the experimental PCS and the back-calculated PCS after tensor refinement (Lescanne et al. 2017). Thus, any change of the Δχ tensors is within the precision of its parameters. The fact that the largest PCS changes map to methyl groups located in the binding site also provides evidence that not a tensor change but movement of the methyl groups is the cause of the PCS changes. Under that assumption, the following calculations were done. We wondered whether the PCS can provide information about the distance range of the rearrangement as well as the new location of the methyl groups. Using the PCS of the bound form, the possible new positions can be calculated as an iso-surface of the new PCS around the lanthanoid. By determining the cross-sections of such iso-surfaces from two or even three mutants, the location space can be reduced, as was shown above for the free protein.

**Table 2:** Significant ΔPCS (ppm). Methyl groups for which two |ΔPCS| > 0.04 ppm are listed

	50C/54C	101C/105C	149C/187C
L48_δ2_	− 0.046	− 0.058	No data
L103_δ1_	− 0.032	− 0.065	0.064
L107_δ2_	0.097	No data	0.067
V136_γ1_	0.091	0.039	− 0.046

**Fig. 9:**
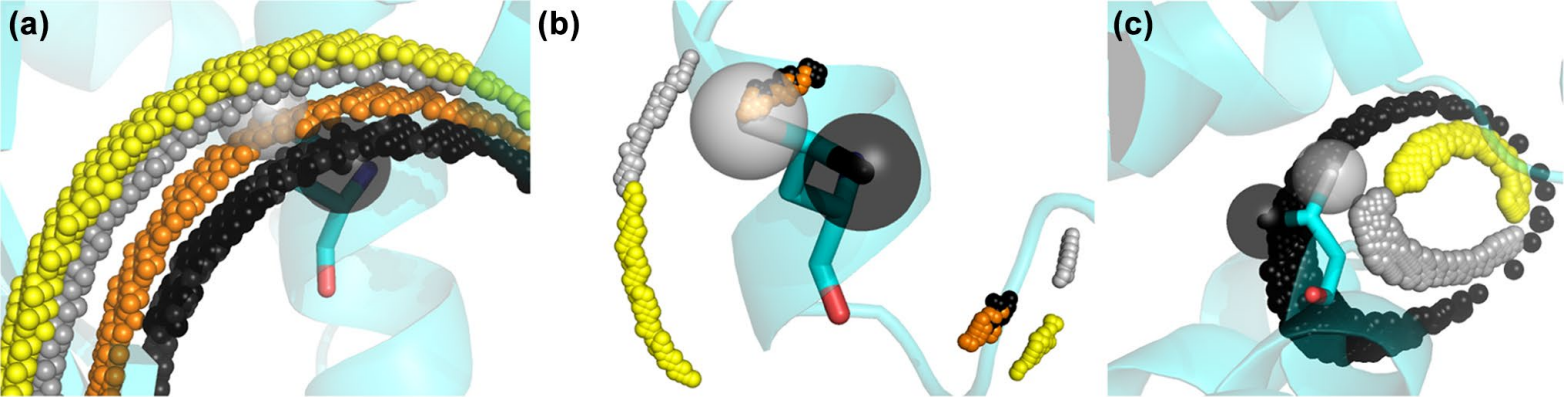
Positions for methyl groups in the ntd-HSP90 bound to** 1**. Small spheres represent PCS iso-surfaces cross-sections in the free state, grey for Valγ1/Leuδ1 and black for Valγ2/Leuδ2, and in the bound state, yellow for Valγ1/Leuδ1 and orange for Valγ2/Leuδ2. The residues as found in the crystal structure of the free protein (PDB entry 3t0h) are shown in cyan sticks, with the methyl groups in the analogous colors shown as 0.7 Å radius spheres centred on the carbon methyl.** a** Leu48, with cross-sections for PCS from mutants 50C/54C and 101C/105C. The minimal distance from the crystal structure conformation to the free cross-sections is 0.8 Å (δ1) and 0.6 Å (δ2), and to the bound cross-sections is 1.3 Å (δ1) and 1.2 Å (δ2).** b** Leu103, the yellow and orange spheres represent the triple cross-sections of mutant 50C/54C with 101C/105C and 149C/187C. The grey and black spheres represent the triple-mutant cross-sections for the free state, at a distance of 1.6 Å (δ1) and 2.3 Å (δ2) from the crystal structure positions. Note that the orange and black region overlap whereas the grey and yellow are bordering, suggesting that methyl δ1 moves, whereas δ2 does not.** c** Val136, the grey and yellow small spheres represent the triple cross-section for methyl group γ1 in free and bound state respectively. The small black spheres represent the free double cross-section between mutant 50C/54C and 101C/105C of group γ2. The orange cross-section is not shown because the γ2 methyl group showed insignificant PCS changes

All cross-sections for the ligand bound state could be determined using an uncertainty of 0.02 ppm, except for Leu107, for which the uncertainty had to be raised to 0.03 ppm to find a cross-section. The cross-sections are shown in Fig. 9 in grey (γ1/δ1) and black (γ2/δ2) for the PCS of free protein and in yellow (γ1/δ1) and orange (γ2/δ2) for the PCS observed for the bound state. For Leu48, iso-surfaces could be calculated for mutants 50C/54C and 101C/105C and the cross-sections are shown for the δ1 and δ2 methyl groups, Fig. 9a. The conformation of the Leu in the free protein is shown in sticks. The Leu methyl groups need to move at least 1.3 Å to move into the cross-section area of the bound state. Similarly, Fig. 9b shows the cross-sections for Leu103. In this case, data from all three mutants result in only two possible positions for the Leu that can match simultaneously the PCS of both methyl groups to the experimental ones. The center of the triple mutant cross-section area for the δ1 methyl is shifted by 3 Å upon binding of the ligand, whereas for the δ2 methyl it does not change, which could indicate that the sidechain rotates. Similarly, the γ1 methyl group of Val136 experiences a significant change in PCS, whereas the γ2 methyl group does not. The cross-section center of the γ1 methyl group for mutant 50C/54C with mutant 101C/105C shifted 1.2 Å from the position in the free protein. In Fig. 9c, it can be that the cross-section area for the bound state (yellow) has moved relative to the one for the free state (light gray). For Leu107, the two iso-surfaces, from mutants 50C/54C and 149C/187C are similar because the two tensors happen to be nearly parallel (Lescanne et al. 2017). Consequently, the cross-section is a curve area rather than a line like in the cases discussed above (Fig. S8). This cross-section area in the bound state is at a large distance from the conformation of Leu107 in the crystal structure of the free protein, the minimal distance between the δ2 methyl group and the cross-section being more than 5 Å, while it is only 0.6 Å for the free state. Thus, a large change in position is suggested by the observed change in the PCS. In different crystal structures of ntd-HSP90, Leu 107 is found in very different orientations, with 3.0 Å between Cα atoms in the structure of the free protein [PDB entry 3t0h (Li et al. 2012)] and the one with ntd-HSP90 bound to a close analogue of **1 [**PDB entry: 2xdk (Murray et al. 2010)]. This observation suggests that Leu107 is very sensitive to ligand binding. Therefore, the observed change in methyl group position is plausible, although it should be treated with caution because of the high degree of correlation between the two Δχ tensors involved. The data for mutant 101C/105C cannot be used in this case because the tag is too close to this Leu 107.

In conclusion, the small but significant changes in the PCS yield consistent information about the change in methyl group position that can be in the range of 1–5 Å. The iso-surface approach yields a limited set of possible conformations, in particular in the case when data from three tag locations are available. These data could be translated into distance restraints, for example for ligand docking studies.

## Conclusions

Methyl groups are widely used NMR probes but the applications of PCS to study them remain limited (Brewer et al. 2015). A straightforward application of paramagnetic tagging is the additional dispersion induced by PCS. Paramagnetic tags have already been used to disperse resonances of an intrinsically disordered protein, presenting a crowded amide proton spectrum (Gobl et al. 2016). Our data demonstrate again that additional dispersion is also relevant for folded, larger proteins that are being studied with methyl group NMR probes, because two-dimensional spectra usually show considerable crowding for methyl resonances in the ^1^H region of 0.8–1.0 ppm, as was reported and used before (Sattler and Fesik 1997). Ytterbium was the lanthanoid of choice for a 25 kDa protein. For larger proteins, lanthanoid of different paramagnetic ‘strength’ could be used to benefit from a high dispersion, while limiting loss of information because of PRE.

Furthermore, PCS were used to provide evidence for small-scale movements (1–3 Å) of methyl groups upon ligand binding, by using a triangulation approach. This makes the PCS a powerful tool to observe re-arrangement of the side chains solely on the basis of 2D NMR spectra. Of course, assignments are required for the interpretation of such data. Recently, we have demonstrated that the same paramagnetic constructs used in the ligand titrations could be used to obtain partial assignments of the methyl groups (Lescanne et al. 2017). Thus, this application of PCS could complement the use of NOESY experiments, or indeed crystal structures, for structure determination of ligand–protein complexes. An advantage is that for measurement of PCS only low sample concentrations are required. It is clear that a single paramagnetic center is not sufficient to define a relevant area for the methyl group position in the bound state. With two paramagnetic centers and the use of both methyl groups of valine and/or leucine the positions can be approximated, because of the additional steric constraint that the two methyl groups must be at a distance of 2.5 Å. With three tags, the location becomes quite restrained, although two or three quite different positions may be found due to the shape of PCS iso-surfaces. Attention should be paid to the relative orientations of the susceptibility tensors used to generate the PCS. In case of tensors with parallel main axes, cross-intersections of iso-surfaces are less resolved or non-existing. When using PCS for methyl localization, it is important to realize that the PCS is highly anisotropic and falls off with the third power of the distance between the nucleus and the paramagnetic center. Localized, high PCS gradients make the PCS very sensitive to the methyl group position. That is an advantage but also a danger. The higher the PCS gradient, the larger the effect of any PCS error will be. The use of several tag positions can counteract this problem. However, this approach also demonstrated that positions predicted from PCS from two or three tags do not always match perfectly with those observed in the crystal state of the protein, even in the absence of ligand. The reasons are not clear. It could be that the average position of these methyl groups is slightly different in the solution state but it is also possible that (one of) the tags have subtle structural effects causing a small mismatch between PCS of the native and tagged protein variants. Consequently, to study the effects of ligand binding it is recommended to use the difference in PCS between free and bound states to derive distance restraints for methyl movement, as was discussed above. To obtain reliable PCS the use of a probe that is rigid relative to the protein is important. The high rigidity of two-armed CLaNP-5 enables the measurement of accurate PCS and the prediction of the tensors parameters accurately. The choice of the lanthanoid determines the optimal region. Nuclei too close to the tag will experience PRE, nuclei too far experience small changes in PCS upon displacement. With Yb^3+^ used here, distances between nucleus and tag in the range of 50 Å yielded PCS changes of 0.04–0.1 ppm that appear to match with displacements of 1–3 Å. The ability to observe and characterize small but significant changes in the methyl group positions can potentially also be applied to large proteins, because it is well-established that with deuteration methyl groups can be detected in very large systems (Saio et al. 2014; Kerfah et al. 2015). We also note that this approach is not limited to ligands that are in fast exchange. As long as assignments are available for free and bound states of the protein, PCS can also be obtained in slow-exchange systems.

## Electronic supplementary material

Below is the link to the electronic supplementary material.

Supplementary material 1 (DOCX 5228 KB)

## Abbreviations

PCS: Pseudocontact shift
HSQC: Heteronuclear single quantum coherence
TROSY: Transverse relaxation optimized spectroscopy
CLaNP-5: Caged lanthanoid NMR probe 5
ntd-HSP90: N-terminal domain of heat shock protein 90
