# Supplementary material for: Methyl group reorientation under ligand binding probed by pseudocontact shifts
Source: J Biomol NMR. 2018 Jun 2;71(4):275–85. doi: 10.1007/s10858-018-0190-5 (PMC6132577; doi:10.1007/s10858-018-0190-5)
Supplement: Supplementary file 1 — Supplementary material 1 (DOCX 5228 KB) [file 10858_2018_190_MOESM1_ESM.docx]

Supplementary information to

**Methyl group reorientation under ligand binding probed by pseudocontact shifts**

Mathilde Lescanne, Puneet Ahuja, Anneloes Blok, Monika Timmer, Tomas Akerud, Marcellus Ubbink

**Table S1.** Nuclei used to refine K_D_ and k_OFF_ with TITAN.

| Mutant |  |  |
| --- | --- | --- |
| 50C/54C | Lu^3+^ | L107δ1, L103δ2, V186γ2, L198δ1, L29δ2 |
| 50C/54C | Yb^3+^ | L107δ1, L103δ2, V186γ2, L198δ1, L29δ2 |
| 101C/105C | Lu^3+^ | V172γ1, L48δ1, L48δ2, L29δ1, L29δ2 |
| 101C/105C | Yb^3+^ | V172γ1, L48δ1, L48δ2, L29δ1, L29δ2 |
| 149C/187C | Yb^3+^ | L103δ1, L103δ2, V186γ2, L45δ1, L29δ2 |
| 149C/187C | Lu^3+^ | L103δ1, L103δ2, V186γ2, L45δ1, L29δ2 |
| WT | no tag | L45H^N^, S140H^N^, F134H^N^, Y160H^N^ |

**Table S2**. PCS measured on free and bound protein for the mutants 50C/54C, 101C/105C and 149C/187C.

| Residue number | stereo | 50C/54C | | | 101C/105C | | | 149C/187C | | |
| --- | --- | --- | --- | --- | --- | --- | --- | --- | --- | --- |
|  |  | Free PCS | Bound PCS | ∆PCS | Free PCS | Bound PCS | ∆PCS | Free PCS | Bound PCS | ∆PCS |
| 17 | HG1 | 0.045 | 0.03 | -0.015 | 0.208 | 0.22 | 0.012 | 0.104 | 0.077 | -0.027 |
| 17 | HG2 | 0.045 | 0.032 | -0.013 | 0.145 | 0.124 | -0.021 | 0.05 | 0.058 | 0.008 |
| 29 | HD1 | -0.406 | -0.402 | 0.004 | 0.137 | 0.144 | 0.007 | 0.246 | 0.225 | -0.021 |
| 29 | HD2 | -0.465 | -0.483 | -0.018 | 0.217 | 0.257 | 0.04 | No data | No data | No data |
| 32 | HD1 | -0.187 | -0.181 | 0.006 | 0.055 | 0.065 | 0.01 | 0.112 | No data | No data |
| 32 | HD2 | -0.186 | -0.191 | -0.005 | 0.062 | 0.07 | 0.008 | No data | No data | No data |
| 45 | HD1 | 0.329 | 0.305 | -0.024 | 0.102 | 0.097 | -0.005 | 0.319 | 0.338 | 0.019 |
| 45 | HD2 | 0.458 | 0.431 | -0.027 | 0.121 | 0.12 | -0.001 | 0.491 | 0.488 | -0.003 |
| 48 | HD1 | 0.774 | 0.717 | -0.057 | 0.23 | 0.224 | -0.006 | 1.484 | No data | No data |
| 48 | HD2 | 0.565 | 0.519 | -0.046 | 0.284 | 0.226 | -0.058 | 0.553 | No data | No data |
| 56 | HD1 | 1.289 | No data | No data | 0.067 | 0.033 | -0.034 | 0.165 | 0.16 | -0.005 |
| 56 | HD2 | 0.838 | 0.863 | 0.025 | 0.021 | -0.011 | -0.032 | 0.103 | 0.102 | -0.001 |
| 64 | HD1 | -0.215 | -0.183 | 0.032 | -0.114 | -0.152 | -0.038 | 0.069 | No data | No data |
| 64 | HD2 | -0.142 | -0.113 | 0.029 | -0.101 | -0.134 | -0.033 | 0.016 | 0.019 | 0.003 |
| 70 | HD1 | 0.112 | 0.127 | 0.015 | -0.058 | -0.036 | 0.022 | -0.011 | -0.029 | -0.018 |
| 70 | HD2 | 0.197 | 0.216 | 0.019 | -0.081 | -0.125 | -0.044 | -0.008 | -0.009 | -0.001 |
| 76 | HD1 | 0.28 | 0.312 | 0.032 | -0.035 | -0.082 | -0.047 | 0.035 | 0.036 | 0.001 |
| 76 | HD2 | 0.415 | 0.434 | 0.019 | -0.023 | -0.068 | -0.045 | 0.018 | -0.026 | 0.008 |
| 80 | HD1 | 0.296 | 0.272 | -0.024 | 0.110 | 0.104 | -0.006 | No data | No data | No data |
| 80 | HD2 | 0.265 | 0.278 | 0.013 | 0.083 | 0.065 | -0.018 | No data | 0.00 | No data |
| 89 | HD1 | No data | No data | No data | No data | No data | No data | No data | No data | No data |
| 89 | HD2 | 0.268 | 0.249 | -0.019 | 0.123 | 0.125 | 0.002 | 0.125 | 0.141 | 0.016 |
| 92 | HG1 | 0.409 | 0.409 | 0.00 | 0.151 | 0.136 | -0.015 | 0.035 | 0.038 | 0.003 |
| 92 | HG2 | 0.023 | 0.019 | -0.004 | 0.021 | 0.014 | -0.007 | 0.068 | 0.078 | 0.010 |
| 103 | HD1 | 0.203 | 0.171 | -0.032 | 1.491 | 1.426 | -0.065 | 0.402 | 0.477 | 0.064 |
| 103 | HD2 | 0.204 | 0.199 | -0.005 | 1.445 | 1.429 | -0.016 | 0.579 | 0.6 | 0.021 |
| 107 | HD1 | 0.215 | 0.170 | -0.045 | 0.715 | No data | No data | 0.958 | No data | No data |
| 107 | HD2 | 0.217 | 0.120 | -0.097 | 0.998 | No data | No data | 0.849 | 0.782 | -0.067 |
| 122 | HD1 | -0.31 | -0.292 | 0.018 | 0.069 | 0.065 | -0.004 | 0.149 | 0.130 | -0.019 |
| 122 | HD2 | -0.303 | -0.280 | 0.023 | 0.057 | 0.085 | 0.028 | 0.158 | 0.112 | -0.046 |
| 136 | HG1 | -0.646 | -0.737 | -0.091 | 0.283 | 0.322 | 0.039 | 0.265 | 0.219 | -0.046 |
| 136 | HG2 | -1.501 | No data | No data | 0.187 | 0.200 | 0.013 | 0.252 | 0.256 | 0.004 |
| 143 | HD1 | -0.121 | -0.138 | -0.017 | 0.107 | 0.122 | 0.015 | 0.224 | 0.216 | -0.008 |
| 143 | HD2 | -0.129 | -0.162 | -0.033 | 0.119 | 0.124 | 0.005 | 0.292 | 0.282 | -0.010 |
| 144 | HG1 | 0.04 | 0.016 | -0.024 | 0.112 | 0.117 | 0.005 | 1.355 | 1.325 | -0.030 |
| 144 | HG2 | 0.118 | 0.083 | -0.035 | 0.150 | 0.151 | 0.001 | 1.754 | No data | No data |
| 148 | HG1 | 0.15 | 0.122 | -0.028 | 0.298 | 0.305 | 0.007 | -0.106 | No data | No data |
| 148 | HG2 | 0.25 | 0.224 | -0.026 | 0.383 | 0.435 | 0.052 | 0.630 | No data | No data |
| 150 | HG1 | 0.356 | 0.321 | -0.035 | 0.655 | 0.659 | 0.004 | 0.205 | No data | No data |
| 150 | HG2 | 0.785 | 0.849 | 0.064 | No data | No data | No data | 1.080 | No data | No data |
| 172 | HG1 | 0.146 | 0.134 | -0.012 | 1.268 | 1.195 | -0.073 | No data | No data | No data |
| 172 | HG2 | 0.065 | 0.043 | -0.022 | No data | No data | No data | 0.209 | 0.198 | -0.011 |
| 186 | HG1 | 0.203 | 0.162 | -0.041 | 0.119 | 0.097 | -0.022 | No data | No data | No data |
| 186 | HG2 | 0.719 | 0.689 | -0.030 | 0.377 | 0.373 | -0.004 | 1.625 | 1.614 | -0.011 |

**Table S3.** Δχ tensor parameters calculated with PCS from the free and bound states of ntd-HSP90, using the 3T0Z structure. Errors are given between brackets.

| **Tensor** | **χ_ax_** | **χ_rh_** | **x** | **y** | **z** | **α** | **β** | **γ** | **Qa** |
| --- | --- | --- | --- | --- | --- | --- | --- | --- | --- |
| **50C/54C free** | 8.3 (0.5) | 2.4 (1.3) | 12.6 (0.8) | 0.4 (0.7) | 25.2 (0.6) | 164 (6) | 154 (5) | 146 (10) | 0.072 |
| **50C/54C bound** | 7.9 (0.3) | 1.5 (0.9) | 12.8 (0.7) | -0.1 (0.6) | 24.9 (0.4) | 157 (4) | 150 (4) | 136 (7) | 0.046 |
| **101C/105C free** | 7.6 (0.3) | 2.9 (0.8) | 24.9 (0.3) | -10.1 (0.4) | 6.5 (0.5) | 176 (3) | 85 (3) | 160 (8) | 0.022 |
| **101C/105C bound** | 8.5 (0.5) | 3.0 (1.0) | 25.8 (0.3) | -8.3 (0.7) | 6.1 (0.7) | 5 (5) | 98 (4) | 35 (12) | 0.026 |
| **149C/187C free** | 9.7 (1.8) | 4.0 (2.9) | -1.1 (1.2) | -4.4 (1.5) | -2.2 (1.2) | 160 (21) | 156 (5) | 42 (16) | 0.115 |
| **149C/187C bound** | 7.9 (1.5) | 2.9 (1.5) | 1.3 (1.3) | -6.1 (1.7) | -2.0 (1.0) | 150 (18) | 157 (7) | 31 (14) | 0.139 |

| 188 | HD1 | 0.27 | 0.231 | -0.039 | 0.223 | 0.228 | 0.005 | 0.302 | 0.338 | 0.036 |
| --- | --- | --- | --- | --- | --- | --- | --- | --- | --- | --- |
| 188 | HD2 | 0.314 | 0.295 | -0.019 | 0.191 | 0.199 | 0.008 | 0.265 | no data | no data |
| 190 | HD1 | 0.131 | 0.109 | -0.022 | 0.130 | 0.135 | 0.005 | 0.292 | 0.302 | 0.010 |
| 190 | HD2 | 0.123 | 0.102 | -0.021 | 0.112 | 0.104 | -0.008 | -0.187 | -0.192 | -0.005 |
| 198 | HD1 | 0.138 | no data | no data | 0.081 | 0.088 | 0.007 | -0.111 | -0.114 | -0.003 |
| 198 | HD2 | 0.094 | 0.076 | -0.018 | 0.097 | 0.093 | -0.004 | -0.047 | -0.054 | -0.007 |
| 207 | HG1 | 0.262 | 0.257 | -0.005 | 0.055 | 0.053 | -0.002 | 0.132 | 0.136 | 0.004 |
| 207 | HG2 | 0.191 | 0.199 | 0.008 | 0.069 | 0.070 | 0.001 | 0.320 | 0.271 | -0.049 |
| 220 | HD1 | 0.143 | 0.138 | -0.005 | 0.059 | no data | no data | 0.094 | 0.107 | 0.013 |
| 220 | HD2 | 0.181 | 0.175 | -0.006 | 0.069 | 0.062 | -0.007 | -0.769 | no data | no data |
| 222 | HG1 | 0.092 | 0.090 | -0.002 | 0.006 | 0.014 | 0.008 | -0.175 | no data | no data |
| 222 | HG2 | 0.96 | 0.088 | -0.008 | 0.050 | 0.050 | 0.000 | -0.230 | no data | no data |


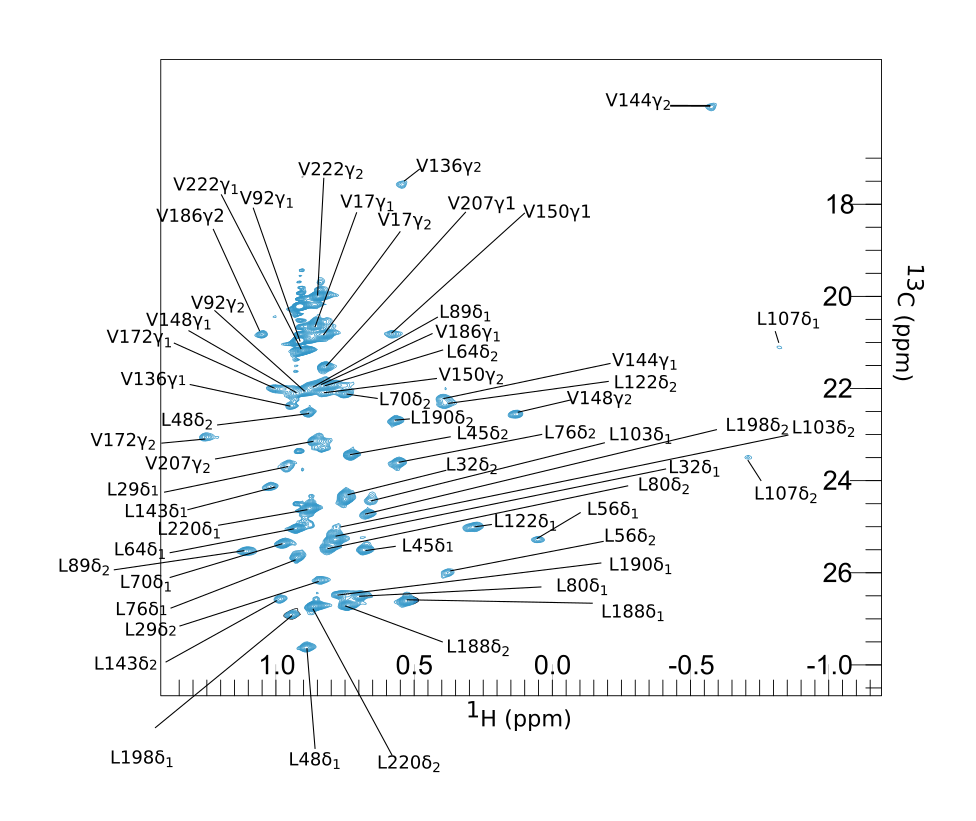


**Figure S1** ^13^C Leu/Val methyl group spectrum of the 101C/105C CLaP5-Lu^3+^ tagged ntd-HSP90.

**Figure S2** Quality of Δχ fits based on amide PCS [Lescanne *et al.* (2017) J. Biomol. NMR 69, 183]. The calculated PCS are plotted against the observed PCS of amide groups of ntd-HSP90 tagged with Yb^3+^-CLaNP-5 at the indicated positions. The solid lines represent a perfect correlation (x=y). Q/Q_a_ values, 0.056/0.028 (50/54C), 0.068/0.034 (101C/105C), 0.26/012 (149C/187C).

**
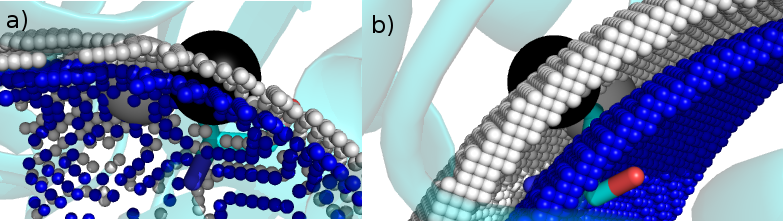
**

**Figure S3**. PCS gradient effects. In light grey are the iso-surfaces of the predicted PCS, in blue are the iso-surfaces of the experimental PCS, for Val136 γ2, represented as a black sphere. All iso-surfaces were calculated with an uncertainty of 0.01 ppm, for clarity. a) Iso-surfaces for mutant 50C/54C, the difference between the two surfaces (0.6 Å) represents a PCS difference of 0.16 ppm. b) Iso-surfaces for mutant 149C/187C, the difference between the two surfaces (1.5 Å) represents a PCS difference of 0.05 ppm.


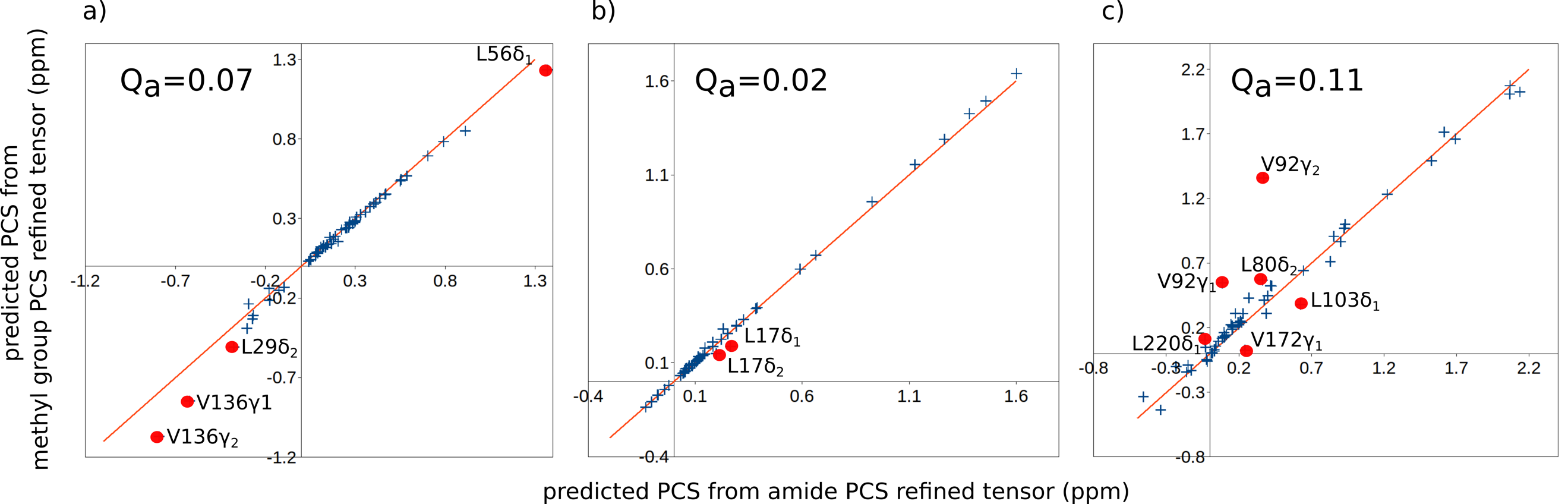


**Figure S4.**  Plot of the predicted PCS calculated with the amide based tensor versus the predicted PCS calculated with the methyl PCS based Δχ tensor for mutant 50C/54C (a), mutant 101C/105C (b) and mutant 149C/187C (c).


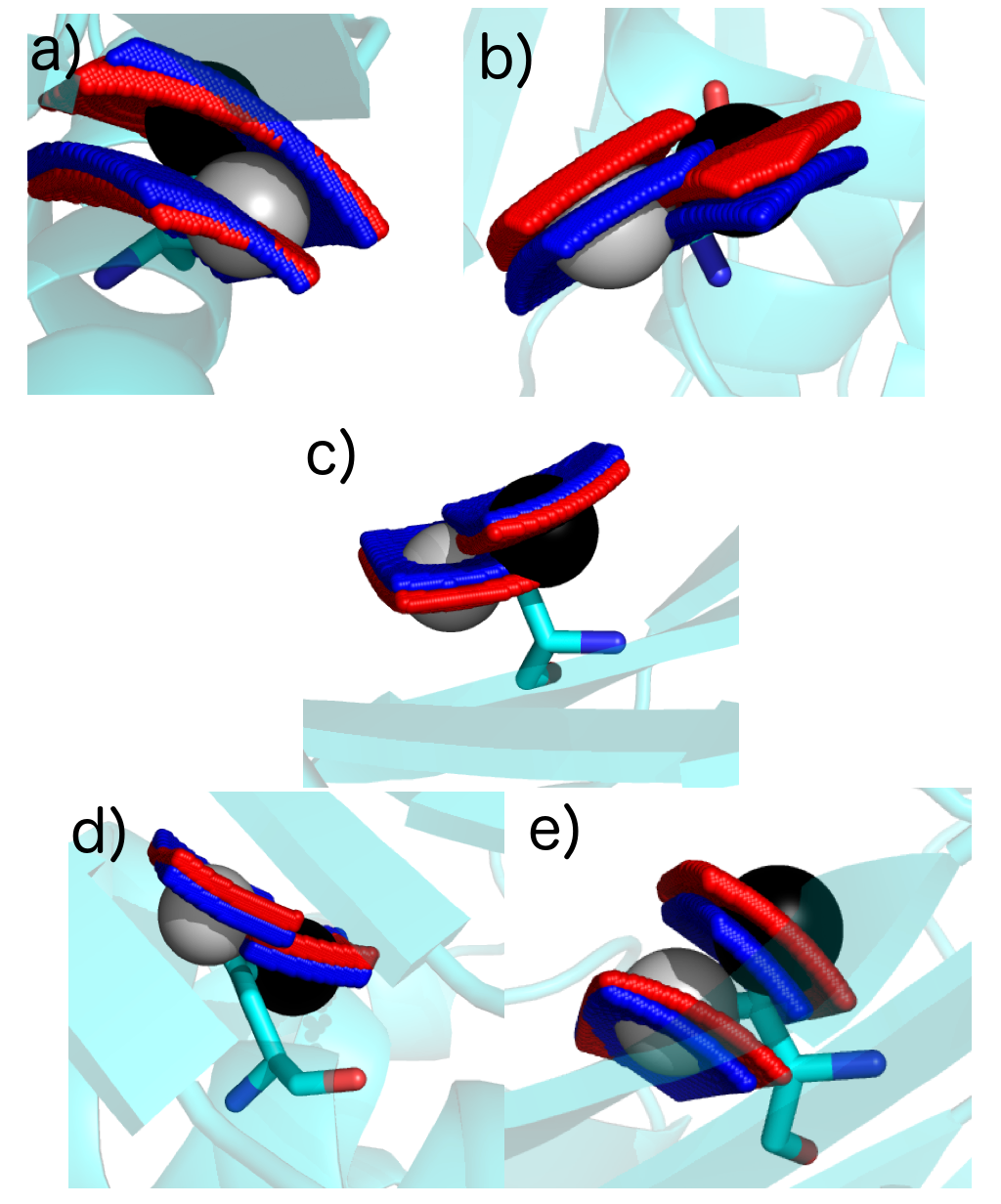


**Figure S5.** Influence of Δχ tensor variation on the iso-surface. Grey and black spheres represent the free carbon δ_1_/γ_1_ and δ_2_/γ_2_ methyl groups (PDB: 3t0h), respectively. The iso-surfaces predicted on the basis of the amide and methyl PCS based Δχ tensors are shown in red and blue, respectively. a) L56 methyl groups, for mutant 50C/54C; b) V136 methyl groups, for mutant 50C/54C; c) V172 methyl groups, for mutant 101C/105C; d) L48 methyl groups, for mutant 149C/187C; e) V148 methyl groups, for mutant 149C/187C.


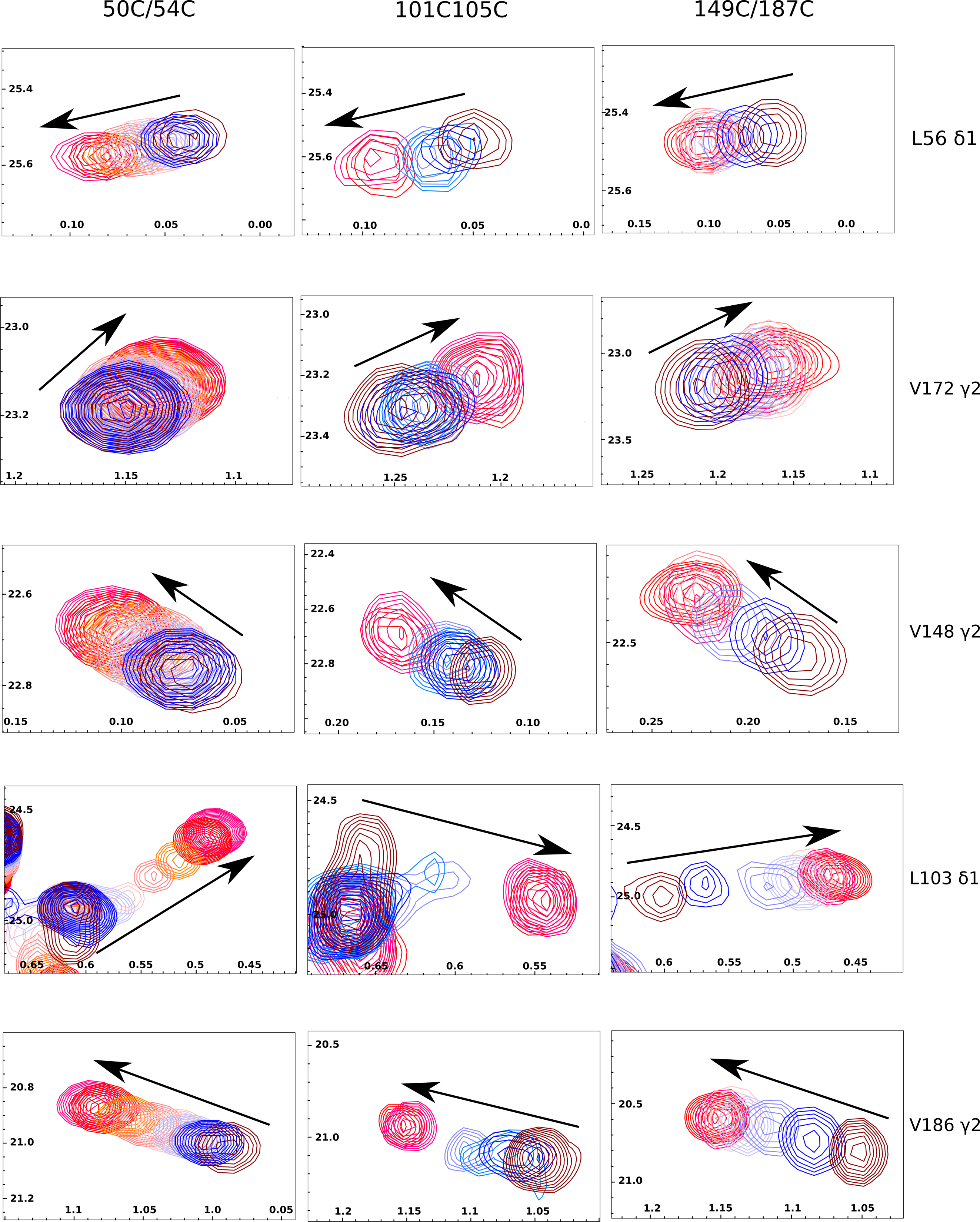


**Figure S6.** CSP patterns. Details of the overlay of the HSQC spectra of ntd-HSP90 titrations with **1** are shown for 5 methyl group resonances. The arrows indicate the direction of shifts. Except for methyl group L103 δ1, the CSP patterns are similar for the three mutants.


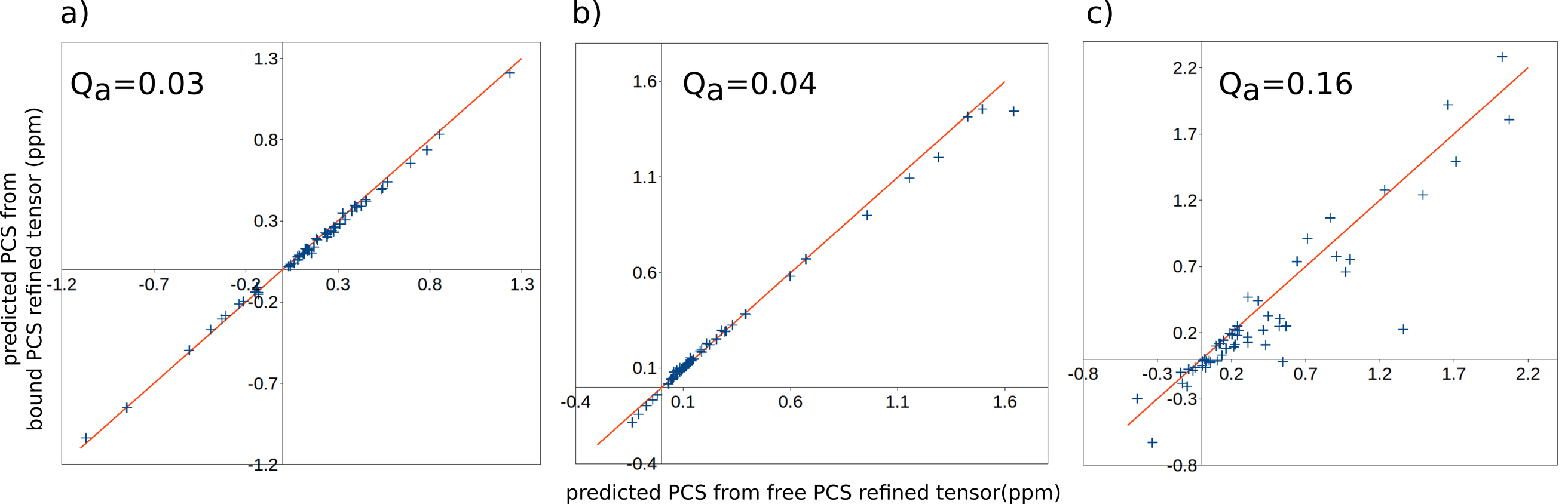


**Figure S7.** Plot of the predicted methyl PCS calculated with Δχ tensors based on the PCS observed of the free (horizontal axis) and bound states (vertical axis), for 50C/54C (a), 101C/105C (b) and 149C/187C (c). The tensor parameters are shown in **Table S3.**


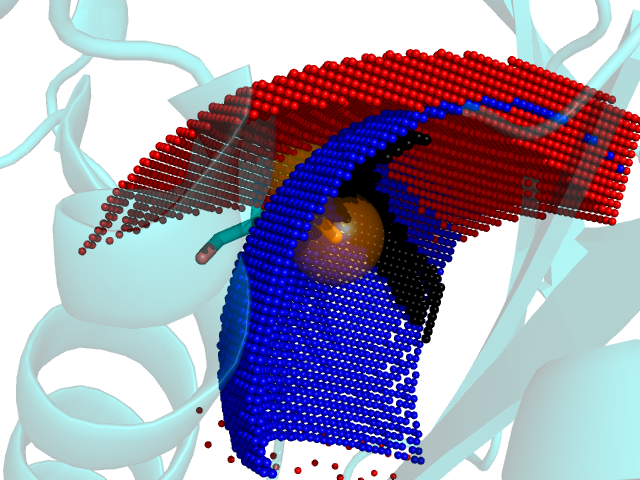


**Figure S8.** Leu107 δ2 methyl group. The blue and red spheres represent the δ2 iso-surfaces for the experimental PCS of the bound state for mutants 50C/54C and 149C/187C, respectively. The cross-section between these two iso-surfaces is obtained using an uncertainty of 0.03 ppm. In the free state, the cross-section between 50C/54C and 149C/187C, in small black spheres, is at a minimum distance of 0.6 Å from the crystal state methyl carbon.
